# Single-cell analysis unravels divergent gene signatures shaping seminoma stemness and metastasis

**DOI:** 10.1038/s41420-025-02802-4

**Published:** 2025-11-07

**Authors:** Zhouliang Bian, Biying Chen, Jiahui Guo, Jiamin Zhang, Guangye Du, Shufang He, Haihua Yuan, Yue Zhou, Bin Jiang, Daliu Min, Dingwei Ye, Hengchuan Su, Yanjie Zhang

**Affiliations:** 1 Department of Oncology, Ninth People’s Hospital, Shanghai Jiao Tong University School of Medicine, Shanghai, PR China; 2 Shanghai Institute of Precision Medicine, Ninth People’s Hospital, Shanghai Jiao Tong University School of Medicine, Shanghai, PR China; 3 Department of Oncology, Shanghai Jiao Tong University Affiliated Sixth People’s Hospital, Shanghai, PR China; 4 Department of Pathology, Ninth People’s Hospital, Shanghai Jiao Tong University School of Medicine, Shanghai, PR China; 5 School of Biomedical Engineering, Shanghai Jiao Tong University, Shanghai, PR China; 6 Department of Urology, Fudan University Shanghai Cancer Center, Shanghai, PR China; 7 Department of Oncology, Shanghai Medical College, Fudan University, Shanghai, PR China

**Keywords:** Testicular cancer, Metastasis, RNA sequencing

## Abstract

Seminoma is the most common solid malignant tumor of the testis in young males, significantly impacting fertility. Approximately 20% of seminomas metastasize, markedly increasing recurrence risk and compromising quality of life. To investigate the poorly understood mechanisms driving seminoma metastasis, we performed a comprehensive analysis by integrating single-cell RNA sequencing, TCGA data mining, and cell biology assays. We identified significant intratumoral heterogeneity. A subset of tumor cells expressing *DPPA4* and *PSMA7* showed high stemness, enhanced self-renewal, and association with metastasis. Knockdown of these genes reduced sphere formation, tumor migration and proliferation in Tcam-2 and NCCIT cells. Conversely, tumor cells overexpressing *PAGE5* and *SAT1* exhibited reduced stemness, migratory capacity and proliferation. In the immune microenvironment, we identified *IFNG*+ T cells, which recruit and activate other antitumor immune cells. These cells secrete IFN-γ, which promotes tumor differentiation, reduces stemness, and mitigates tumor aggressiveness. Based on these findings, we further developed a molecular panel to aid in the identification of seminomas with a higher risk of metastasis. In conclusion, our study identifies unique molecular signatures that facilitate risk stratification based on metastatic potential, providing valuable insights for improving precision medicine.

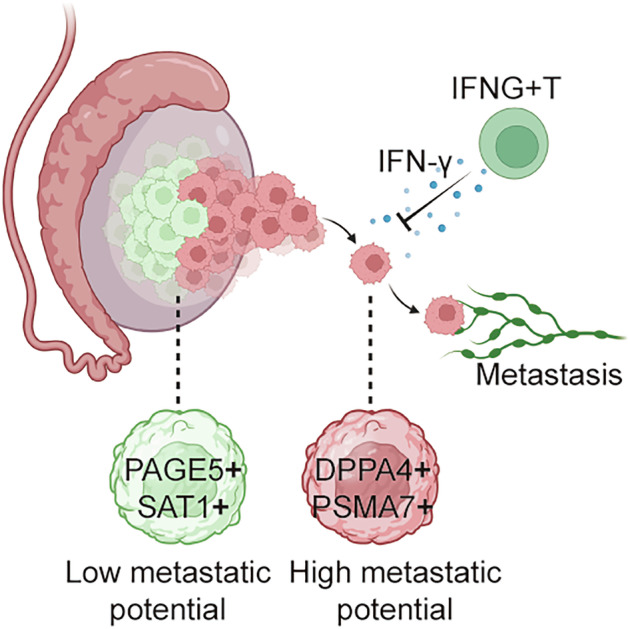

## Introduction

Testicular germ cell tumors (TGCTs) are the most common malignancies among young males, with their incidence doubling over the past four decades [1, 2]. This rise presents a significant challenge to the survival quality and fertility preservation in this patient population [3–6]. TGCTs include seminomas and non-seminomatous germ cell tumors (NSGCTs), with seminomas accounting for approximately 55–60% of all TGCTs [1, 7–9]. Notably, around 80% of seminoma cases are diagnosed at stage I, where survival rates approach 99% with appropriate treatment [1, 10, 11]. Consequently, reducing treatment-associated toxicity and improving long-term health outcomes are critical priorities in these patients [12–14]. However, approximately 20% of seminoma cases metastasize, significantly increasing the risk of recurrence, which necessitates intensified chemotherapy and adversely impacts patients’ quality of life. Accurate molecular classification of seminomas could help clinicians stratify patients with different risk of metastasis and significantly advance the development of precision medicine [15–17].

TGCTs originate from gonocytes, which typically differentiate into spermatogonial stem cells [18–20]. Seminomas retain characteristics similar to gonocytes, while embryonal carcinoma within NSGCTs reflects the characteristics of embryonic stem cells [21]. Previous studies have demonstrated that unipotent gonocytes can be reprogrammed in vitro to acquire pluripotency, characterized by the activation of pluripotency-associated genes [22–24]. However, the differentiation states and associated molecular heterogeneity of seminomas within patients remain poorly understood. This heterogeneity may help explain why 20% of seminoma patients develop metastases, which are associated with a post-surgery recurrence rate of up to 18% [2]. A lack of single-cell RNA sequencing studies has limited our understanding of the molecular drivers of metastatic seminomas.

Previous pathological studies have revealed extensive lymphocyte infiltration in seminomas [25, 26]. However, the mechanisms underlying lymphocyte recruitment and their role remain poorly characterized, especially given the testis’s intrinsic immune-privileged status that protects germ cells from autoimmune attack [27]. Whether seminomas exploit this immune-privileged microenvironment to evade immune surveillance is not yet clear.

In this study, we demonstrate that seminomas exhibit substantial intratumoral heterogeneity: one subpopulation resembles embryonal carcinoma cells with stronger stemness properties and higher malignancy, while the other resembles more differentiated spermatogonia, suggesting a less aggressive phenotype. We further uncover a population of *IFNG*+ T cells within the immune microenvironment of seminomas that suppress metastatic progression. These cells mediate their effect by recruiting additional immune cell subpopulations that promote antitumor immunity. Additionally, IFN-γ secreted by *IFNG*+ T cells acts directly on seminomas, promoting differentiation and thereby inhibiting tumor progression.

Our study identifies unique molecular characteristics, including enhanced stemness, increased migratory potential and heightened self-renewal capacity that distinguish metastatic from non-metastatic seminomas. We developed a molecular panel that identifies patients with high risk of metastasis, guiding the implementation of intensive surveillance, and also recognizes those with low risk of disease progression, allowing for reduced treatment intensity. These findings pave the way for improving precision medicine strategies in seminoma patients.

## Results

### Single-cell analysis reveals distinct cellular subpopulations in seminoma

**Fig. 1:**
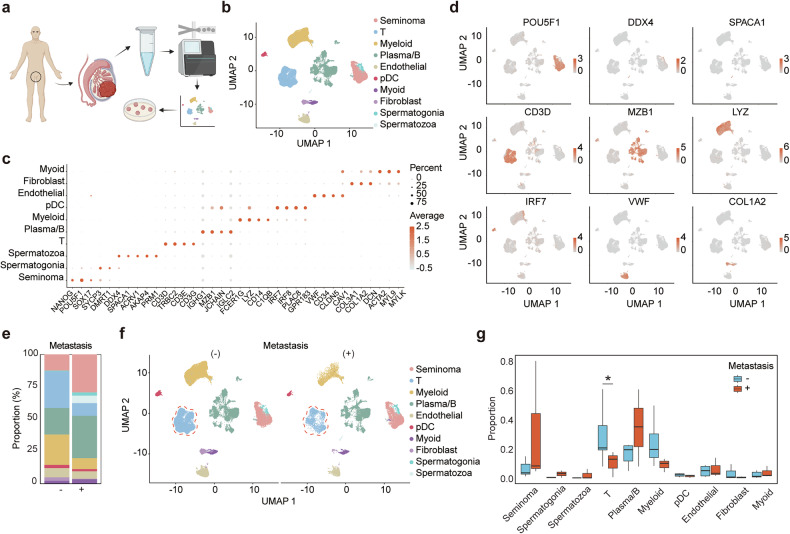
Cellular composition analysis in seminomas. **a** Workflow illustrating sample collection, data analysis, and validation processes (Created in BioRender. Bian, Z. (2025) https://BioRender.com/t10k902). **b** UMAP plot showing cells from seminoma samples, colored by cell population identities. **c** Dot plot depicting the expression levels of canonical marker genes across different cell populations. **d** UMAP visualization of canonical marker gene expression patterns. **e** Bar plot summarizing the cellular composition. **f** UMAP plot highlighting cell clusters in metastatic versus non-metastatic samples. **g** Box plot comparing the proportions of various cell types between metastatic and non-metastatic samples.

To identify molecular features associated with seminoma metastasis, single-cell RNA sequencing (scRNA-seq) was performed on primary seminoma tissues from 7 patients with non-metastatic disease and 3 patients with lymph node and/or distant metastases (Fig. 1a). After filtering out low-quality cells, a total of 73,992 cells were retained, comprising 50,684 cells from the non-metastatic group and 23,308 cells from the metastatic group (Fig. S1). Dimensionality reduction and graph-based clustering identified ten major cellular populations (Fig. 1b).

Using canonical cell type markers, these populations were annotated as follows: seminoma cells (*NANOG*, *POU5F1*, *SOX17*), T cells (*CD3D*, *CD3E*, *CD3G*), myeloid cells (*LYZ*, *FCER1G*, *CD14*), plasma/B cells (*MZB1*, *JCHAIN*, *IGHG1*), endothelial cells (*VWF*, *CD34*, *CLDN5*), plasmacytoid dendritic cells (*IRF7*, *IRF8*, *PLAC8*), myoid cells (*ACTA2*, *MYL9*, *MYLK*), fibroblasts (*COL3A1*, *COL1A2*, *DCN*), spermatogonia (*SYCP3*, *DMRT1*), and spermatozoa (*SPACA1*, *ACRV1*, *AKAP4*) (Fig. 1c). UMAP visualization confirmed the specific expression of these markers within their respective cellular subpopulations, validating the unsupervised clustering’s accuracy in identifying distinct transcriptional profiles (Fig. 1d).

To investigate changes in cell-type proportions between metastatic and non-metastatic samples, the relative abundance of each cellular population in both groups was compared (Fig. 1e–g). Among all subpopulations, T cells exhibited a significant difference in proportion, with a markedly lower abundance in metastatic seminoma samples compared to non-metastatic samples (Fig. 1g).

### Tumor subpopulations within seminoma are associated with metastasis and stemness

Germ cell tumors are characterized by varying differentiation stages. Embryonal carcinoma exhibits strong stemness and resembles embryonic stem cells, while seminoma resembles gonocytes. Gonocytes themselves retain the potential to further differentiate into spermatogonia and spermatocytes. Since embryonal carcinoma’s strong stemness is linked to more aggressive biological behavior, we hypothesized that seminoma may also harbor tumor subpopulations with varying levels of stemness, potentially influencing clinical characteristics.

**Fig. 2:**
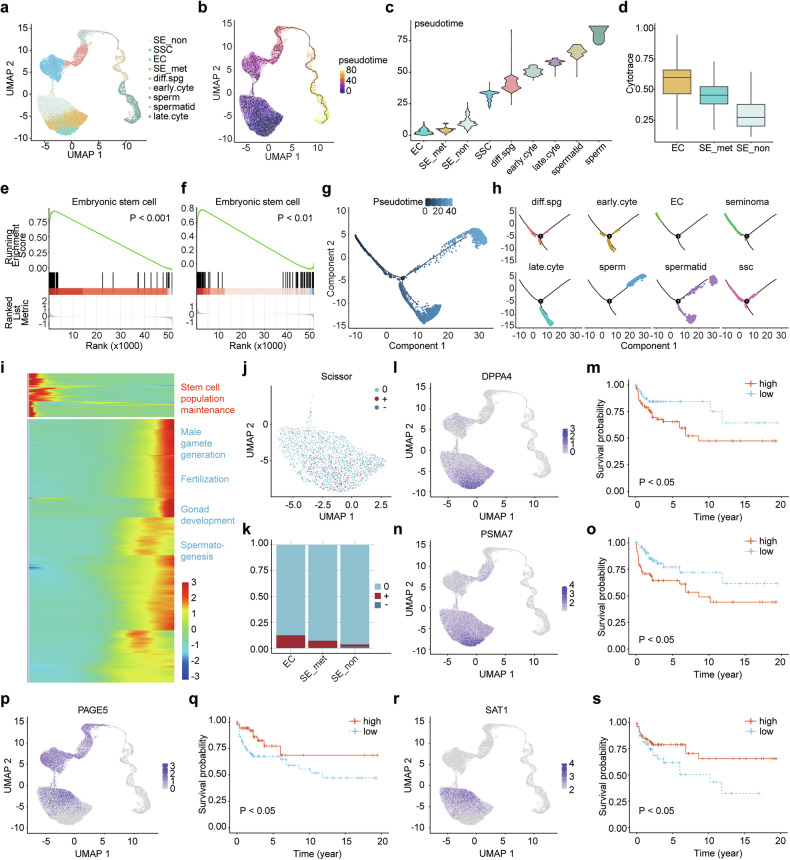
Differentiation states and heterogeneity in seminomas. **a** UMAP plot showing the integration of seminoma cells with embryonal carcinoma and spermatogenesis-related cells (EC embryonal carcinoma, SE_met metastatic seminoma, SE_non non-metastatic seminoma, SSC spermatogonial stem cell, diff.spg differentiating spermatogonia, early.cyte early spermatocyte, late.cyte late spermatocyte). **b** UMAP plot depicting the pseudotime trajectory. **c** Violin plot comparing pseudotime scores across different cell types. **d** Box plot comparing Cytotrace scores among different tumor cell types. **e** GSEA plot showing enrichment of embryonic stem cell-associated signatures in embryonal carcinoma cells compared to seminoma cells. **f** GSEA plot showing enrichment of embryonic stem cell-associated signatures in metastatic seminoma cells compared to non-metastatic seminoma cells. **g** DDRTree plot illustrating the pseudotime trajectory. **h** DDRTree plot highlighting different cell types along the pseudotime trajectory. **i** Heatmap showing genes dynamically expressed along the pseudotime trajectory. **j** UMAP plot highlighting different Scissor groups (“+” indicates cells positively associated with disease progression, “−” indicates cells negatively associated with disease progression, and “0” indicates cells with no association with disease progression). **k** Bar plot summarizing the proportions of Scissor group cells across tumor types. **l** UMAP plot showing *DPPA4* expression. **m** Kaplan–Meier survival plot showing that high *DPPA4* expression correlates with worse progression-free interval (PFI). **n** UMAP plot showing *PSMA7* expression. **o** Kaplan-Meier survival plot showing that high *PSMA7* expression correlates with worse PFI. **p** UMAP plot showing *PAGE5* expression. **q** Kaplan–Meier survival plot showing that high *PAGE5* expression correlates with better PFI. **r** UMAP plot showing *SAT1* expression. **s** Kaplan–Meier survival plot showing that high *SAT1* expression correlates with better PFI.

To investigate the differentiation states of seminoma, seminoma data were integrated with datasets of embryonal carcinoma (high stemness) and spermatogenesis-related cells (high differentiation) (Fig. 2a). Pseudotime analysis using Monocle3 revealed that metastatic seminoma tumor cells were positioned closer to the stemness end of the trajectory compared to non-metastatic tumor cells (Fig. 2b, c). Cytotrace scoring further confirmed that metastatic seminoma cells exhibited higher stemness scores than non-metastatic seminoma cells (Fig. 2d). Transcriptional profiling supported these observations: genes associated with stemness were enriched in embryonal carcinoma cells compared to seminoma cells, while stemness-related gene sets were also enriched in metastatic seminoma cells compared to non-metastatic seminoma cells (Fig. 2e, f). As tumor cells progressed toward a higher degree of malignancy, pathways such as male gamete generation, fertilization, gonad development, and spermatogenesis were progressively downregulated, while pathways like negative regulation of cell differentiation and stem cell population maintenance were increasingly activated (Fig. 2g–i).

We validated the relationship between differentiation states and disease progression using Scissor analysis (Fig. 2j, k). Embryonal carcinoma cells contained the highest fraction of cells associated with progression, followed by metastatic seminoma, whereas non-metastatic seminoma samples had the lowest. Notably, cells linked to a non-progressive state were only observed in non-metastatic seminomas.

**Fig. 3:**
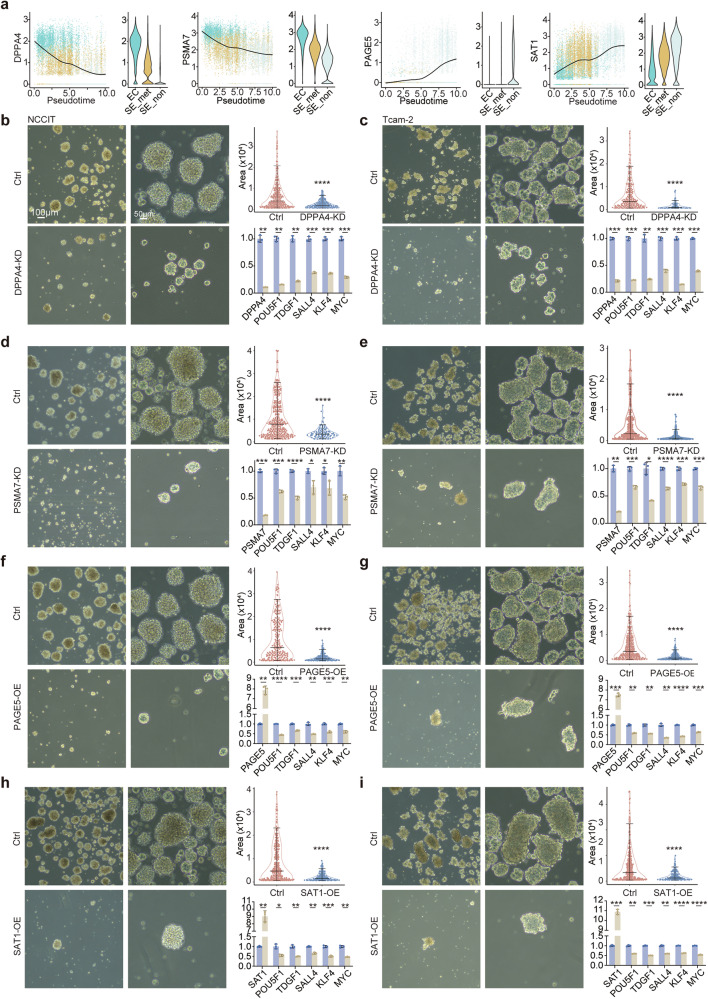
Key genes associated with seminoma stemness. **a** The relationship between stemness-associated genes and pseudotime. **b** Microscopic images of NCCIT spheres after *DPPA4* knockdown (scale bars: 100 µm (left); 50 µm (right)). Violin plot showing reduced sphere size and bar plot indicating decreased expression of stemness-related genes following *DPPA4* knockdown. **c** Microscopic images of Tcam-2 spheres after *DPPA4* knockdown. Violin plot showing smaller spheres and bar plot demonstrating reduced stemness-related gene expression after *DPPA4* knockdown. **d** Microscopic images of NCCIT spheres after *PSMA7* knockdown. Violin plot showing smaller spheres and bar plot indicating reduced stemness-related gene expression following *PSMA7* knockdown. **e** Microscopic images of Tcam-2 spheres after *PSMA7* knockdown. Violin plot showing reduced sphere size and bar plot showing decreased expression of stemness-related genes after *PSMA7* knockdown. **f** Microscopic images of NCCIT spheres after *PAGE5* overexpression. Violin plot showing smaller spheres and bar plot indicating reduced stemness-related gene expression following *PAGE5* overexpression. **g** Microscopic images of Tcam-2 spheres after *PAGE5* overexpression. Violin plot showing smaller spheres and bar plot demonstrating reduced stemness-related gene expression after *PAGE5* overexpression. **h** Microscopic images of NCCIT spheres after *SAT1* overexpression. Violin plot showing reduced sphere size and bar plot showing decreased expression of stemness-related genes following *SAT1* overexpression. **i** Microscopic images of Tcam-2 spheres after *SAT1* overexpression. Violin plot showing smaller spheres and bar plot demonstrating reduced stemness-related gene expression after *SAT1* overexpression.

The analysis identified key genes associated with tumor subsets exhibiting varying levels of stemness and metastatic potential. Genes such as *DPPA4* and *PSMA7* were highly expressed in tumor cells characterized by elevated stemness and enhanced metastatic ability, including embryonal carcinoma and metastatic seminoma, and were linked to disease progression in TCGA analyses (Fig. 2l–o and 3a). In contrast, *PAGE5* and *SAT1*, predominantly expressed in non-metastatic seminoma, were associated with reduced metastatic potential and a lower likelihood of disease progression (Fig. 2p–s and 3a). To further strengthen and validate our conclusions, we analyzed publicly available single-cell RNA sequencing data from paired primary seminoma and matched lymph-node metastasis samples (Fig. S2a) [28]. Consistent with our findings, external dataset demonstrated higher expression of *DPPA4* and *PSMA7* in metastatic lesions compared to primary tumors, while *SAT1* expression was higher in primary sites (Fig. S2b). These expression patterns in metastatic lesions are consistent with the predictions of our model, supporting its robustness and biological relevance. *PAGE5* showed low expression levels in both primary and metastatic tissues, possibly reflecting inter-patient heterogeneity.

Additionally, to confirm whether these transcriptional differences are reflected at the protein level, we performed immunohistochemical (IHC) staining for DPPA4, PSMA7, and SAT1 on tissue sections from our own patient cohort (Fig. S3a). The IHC results revealed protein expression patterns highly consistent with the single-cell transcriptomic data: DPPA4 and PSMA7 protein levels were significantly elevated in metastatic samples, whereas SAT1 and PAGE5 protein levels were relatively higher in non-metastatic primary tumors (Fig. S3b). These concordant results across external datasets, single-cell RNA sequencing, and protein-level validation provide robust evidence supporting the association of these genes with seminoma metastatic potential.

To experimentally validate the roles of these genes in stemness maintenance, sphere formation assays were conducted using the embryonal carcinoma cell line NCCIT and the seminoma cell line Tcam-2 (Fig. 3b–i). Knockdown of *DPPA4* and *PSMA7* significantly reduced the size of tumor spheres in both cell lines (Fig. 3b–e). Similarly, overexpression of *PAGE5* and *SAT1* also dramatically reduced sphere formation (Fig. 3f–i). RT-PCR analysis showed a significant downregulation of stemness-related genes, such as *POU5F1* and *KLF4* in *DPPA4* and *PSMA7* knockdown spheres, as well as *PAGE5* and *SAT1* overexpression spheres (Fig. 3b–i).

**Fig. 4:**
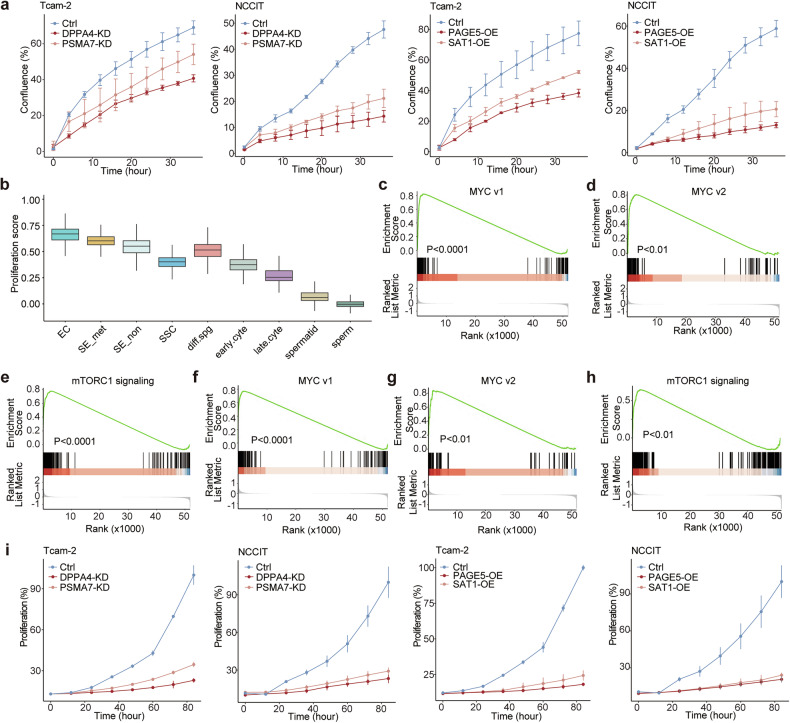
Stemness-associated genes are linked to proliferation in seminomas. **a** Confluence rate of Tcam-2 and NCCIT cells in wound healing assays (Tcam-2: Ctrl vs *DPPA4*-KD: P < 0.01, Ctrl vs *PSMA7*-KD: P < 0.05, Ctrl vs *PAGE5*-OE: P < 0.01, Ctrl vs *SAT1*-OE: P < 0.05; NCCIT: Ctrl vs *DPPA4*-KD: P < 0.001, Ctrl vs *PSMA7*-KD: P < 0.001, Ctrl vs *PAGE5*-OE: P < 0.001, Ctrl vs *SAT1*-OE: P < 0.05). **b** Box plot showing proliferation scores across different cell types. **c** GSEA plot showing enrichment of MYC target v1 gene signature in embryonal carcinoma cells compared to seminoma cells. **d** GSEA plot showing enrichment of MYC target v2 gene signature in embryonal carcinoma cells compared to seminoma cells. **e** GSEA plot showing enrichment of mTORC1 signaling gene signature in embryonal carcinoma cells compared to seminoma cells. **f** GSEA plot showing enrichment of MYC target v1 gene signature in metastatic seminoma cells compared to non-metastatic seminoma cells. **g** GSEA plot showing enrichment of MYC target v2 gene signature in metastatic seminoma cells compared to non-metastatic seminoma cells. **h** GSEA plot showing enrichment of mTORC1 signaling gene signature in metastatic seminoma cells compared to non-metastatic seminoma cells. **i** Growth curves of Tcam-2 and NCCIT cells (Tcam-2: Ctrl vs *DPPA4*-KD: P < 0.01, Ctrl vs *PSMA7*-KD: P < 0.01, Ctrl vs *PAGE5*-OE: P < 0.0001, Ctrl vs *SAT1*-OE: P < 0.0001; NCCIT: Ctrl vs *DPPA4*-KD: P < 0.01, Ctrl vs *PSMA7*-KD: P < 0.01, Ctrl vs *PAGE5*-OE: P < 0.01, Ctrl vs *SAT1*-OE: P < 0.01).

The involvement of these genes in migratory capacity was confirmed through Incucyte 96-well scratch wound migration assay. Knockdown of *DPPA4* and *PSMA7* or overexpression of *PAGE5* and *SAT1* markedly decreased the migratory ability of Tcam-2 and NCCIT cells according to the confluence calculated by the Incucyte Live-Cell Analysis system (Fig. 4a).

As a fundamental property directly linked to self-renewal in stem cells, proliferation capacity was shown to decrease with descending stemness in single-cell data (Fig. 4b). Differential gene expression analysis revealed enrichment of proliferation-related gene sets in embryonal carcinoma cells compared to seminoma cells (Fig. 4c–e). These gene sets were also enriched in metastatic seminoma cells compared to non-metastatic seminoma cells (Fig. 4f–h). Functional experiments demonstrated that knockdown of *DPPA4* and *PSMA7*, as well as overexpression of *PAGE5* and *SAT1*, impaired proliferation in NCCIT and Tcam-2 cells (Fig. 4i). Together, these findings suggest that *DPPA4* and *PSMA7* promote tumor progression, whereas *PAGE5* and *SAT1* suppress progression in germ cell tumors. The identification of these genes provides clues for stratifying patients and optimize treatment strategies.

### Analysis of metastatic and non-metastatic seminoma samples reveals differences in T cells and macrophages

**Fig. 5:**
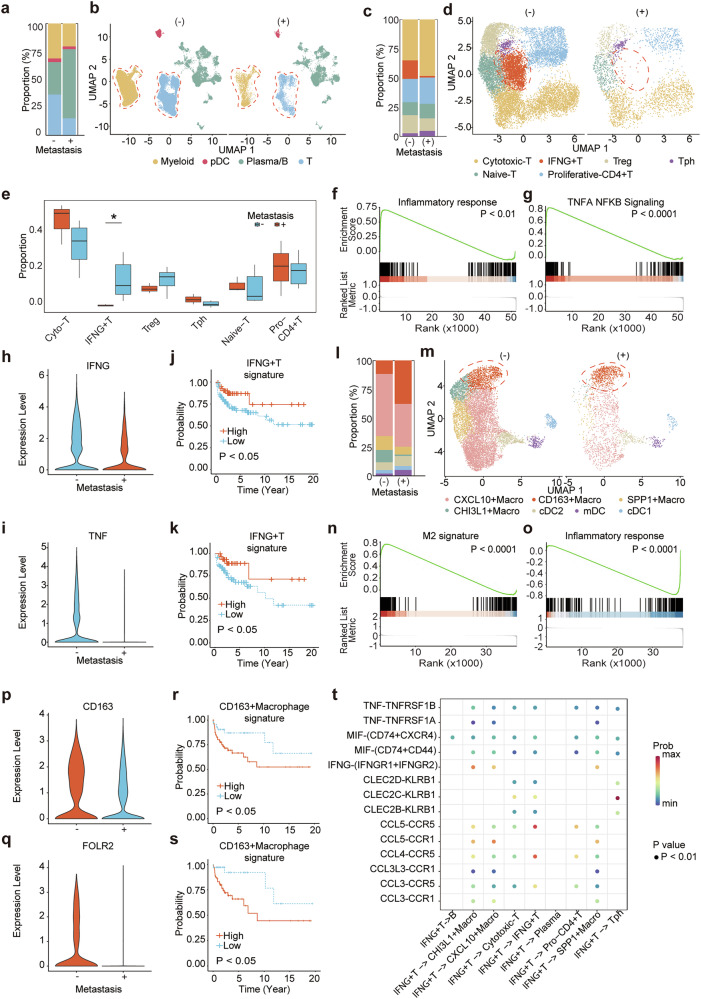
Decoding the immune microenvironment of seminomas. **a** Bar plot summarizing the proportions of immune cell populations. **b** UMAP plot highlighting immune cells in metastatic and non-metastatic samples. **c** Bar plot summarizing the proportions of T cell subpopulations. **d** UMAP plot highlighting T cell subpopulations in metastatic and non-metastatic samples. **e** Box plot comparing T cell subpopulation proportions between metastatic and non-metastatic samples. **f** GSEA plot illustrating the enrichment of an inflammatory response gene signature in non-metastatic T cells. **g** GSEA plot showing the enrichment of the TNF-α/NF-κB signaling gene signature in non-metastatic T cells. **h** Violin plot comparing IFNG expression between metastatic and non-metastatic T cells. **i** Violin plot comparing TNF expression between metastatic and non-metastatic T cells. **j** Kaplan–Meier survival plot showing that the *IFNG*+ T cell-associated signature correlates with better PFI. **k** Kaplan–Meier survival plot showing that the *IFNG*+ T cell-associated signature correlates with better disease-free interval (DFI). **l** Bar plot summarizing the proportions of myeloid cell subpopulations in metastatic and non-metastatic samples. **m** UMAP plot highlighting myeloid subpopulations in metastatic and non-metastatic samples. **n** GSEA plot showing the enrichment of M2 macrophage signatures in *CD163*+ macrophages. **o** GSEA plot showing the enrichment of an inflammatory response gene signature in non-metastatic myeloid cells. **p** Violin plot comparing *CD163* expression between metastatic and non-metastatic myeloid cells. **q** Violin plot comparing *FOLR2* expression between metastatic and non-metastatic myeloid cells. **r** Kaplan–Meier survival plot showing that the *CD163*+ macrophage-associated signature correlates with poorer PFI. **s** Kaplan–Meier survival plot showing that the *CD163*+ macrophage-associated signature correlates with poorer DFI. **t** Bubble plot showing cell–cell interactions in non-metastatic samples.

To further investigate the role of immune cells in the tumor microenvironment of seminoma, we performed additional clustering analysis on immune cells within the dataset (Fig. 5a, b). The proportion of T cells among all immune cells was significantly lower in the metastatic group compared to the non-metastatic group, suggesting that differences in T cell infiltration may be associated with seminoma metastasis (Fig. 5a). To determine the potential roles of different T cell subpopulations, we performed further clustering of all T cells (Fig. 5c, d). This analysis identified six distinct subpopulations: proliferative *CD4*+ T cells, cytotoxic *CD8*+ T cells, naïve T cells, T regulatory cells (Tregs), T peripheral helper (Tph) cells and *IFNG*+ T cells. Each subpopulation was validated by canonical markers (Fig. S4a). A significant difference was only observed in *IFNG*+ T cells, which were less abundant in the metastatic group than in the non-metastatic group (Fig. 5e). Previous studies have established that IFN-γ, a pro-inflammatory cytokine, modulates immune responses and activates cytotoxic immune cells [29]. Other T cell subpopulations showed no significant differences between the groups. Although previous studies have linked cytotoxic *CD8*+ T cells to antitumor responses in seminoma, our data suggest their abundance alone does not distinguish metastatic status [30]. Differential gene expression analysis revealed that inflammatory pathways were significantly enriched in the non-metastatic group (Fig. 5f, g). Furthermore, direct comparison of gene expression between the two groups showed higher expression of inflammation-related genes, including *IFNG* and *TNF*, in the non-metastatic group (Fig. 5h, i). Recognizing the link between strong inflammatory responses and antitumor immunity, we identified an *IFNG*+ T cell gene signature associated with reduced disease progression in the TCGA TGCT dataset, offering a framework for precision therapy (Fig. 5j, k).

Beyond T cells, we observed that the proportion of myeloid cells was lower in the metastatic group compared to the non-metastatic group (Fig. 5a). Further clustering of myeloid cells identified seven subpopulations: *CXCL10+* macrophages, *CD163+* macrophages, *SPP1+* macrophages, *CHI3L1+* macrophages, cDC1, cDC2, and mDC (Fig. 5l, m). Among these, *CD163+* macrophages were significantly more abundant in the metastatic group (Fig. 5l). Notably, although the overall number of macrophages was lower in the metastatic group, the majority were *CD163+* macrophages, which have been linked to M2-like macrophages in previous studies. Transcriptomic analysis of this subpopulation revealed significant enrichment of M2 macrophage-related gene signatures compared to other myeloid subpopulations (Fig. 5n). Given the association between M2 macrophages and anti-inflammatory responses, differential gene expression analysis of myeloid cells showed a marked downregulation of inflammation-related pathways in the metastatic group (Fig. 5o). *CD163* and *FOLR2*, canonical markers of M2 macrophages, were both significantly upregulated in the metastatic group (Fig. 5p, q). Survival analysis based on *CD163+* macrophage-related gene signatures in the TCGA TGCT dataset indicated that higher expression of these genes correlated with increased risk of disease progression (Fig. 5r, s).

Furthermore, our analysis of NSGCT samples demonstrated that these tumors exhibit an immune microenvironment, particularly in the composition of T cells and macrophages, that closely resembles that of metastatic seminomas, potentially reflecting their higher malignant potential (Figs. S4b and 5a, b) [31].

**Fig. 6:**
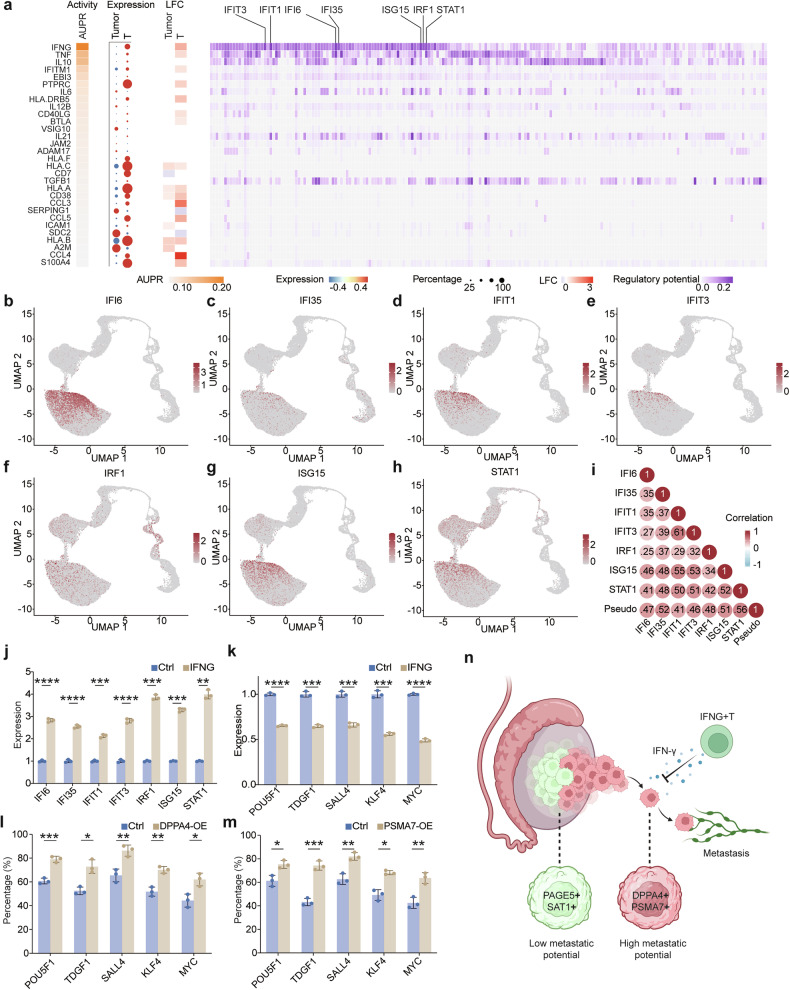
IFN-γ from *IFNG*+ T cells induces seminoma differentiation. **a** NicheNet analysis identifying *IFNG* as a key factor driving differential gene expression between metastatic and non-metastatic seminomas. **b** UMAP plot illustrating the expression of *IFI6* in tumor cells. **c** UMAP plot showing the expression of *IFI35* in tumor cells. **d** UMAP plot showing the expression of *IFIT1* in tumor cells. **e** UMAP plot showing the expression of *IFIT3* in tumor cells. **f** UMAP plot illustrating the expression of *IRF1* in tumor cells. **g** UMAP plot highlighting the expression of *ISG15* in tumor cells. **h** UMAP plot showing the expression of *STAT1* in tumor cells. **i** Correlation dot plot showing positive association among different genes and pseudotime scores in tumor cells. **j** Bar plot showing upregulation of *IFNG*-associated genes following IFN-γ treatment. **k** Bar plot showing downregulation of stemness-associated genes following IFN-γ treatment. **l** Overexpression of *DPPA4* counteracts the differentiation-inducing effect of IFN-γ. **m** Overexpression of *PSMA7* counteracts the differentiation-inducing effect of IFN-γ. **n** Schematic model illustrating the molecular and cellular differences between metastatic and non-metastatic seminomas (Created in BioRender. Bian, Z. (2025) https://BioRender.com/b18k646).

To further elucidate the organizational principles of the seminoma immune microenvironment, we adopted a previously established tumor microenvironment (TME) subtyping framework [32, 33]. Using ssGSEA on pseudobulk single-cell data and TCGA reference data, we classified our samples into four TME subtypes: Immune-Enriched (IE), Fibrotic (F), Desert (D), and IE/F (mixed). Strikingly, most metastatic seminoma samples corresponded to the “Desert” subtype, characterized by minimal immune infiltration and poor response to immunotherapy, while non-metastatic seminomas were enriched for the “Immune-Enriched” subtype, marked by robust immune activation and less aggressive tumor behavior (Fig. 6a). This supports the idea that effective immune surveillance, particularly via *IFNG*+ T cells, is crucial for confining seminomas to the primary site and limiting metastasis, whereas an immune desert microenvironment facilitates tumor progression.

To investigate how different cell subpopulations interact within the tumor microenvironment to influence metastasis, we conducted a CellChat interaction analysis (Figs. 5t and S6b, c). The analysis revealed that *IFNG*+ T cells secrete IFN-γ and TNF, which act on macrophages to enhance their cytotoxic and antigen-presenting functions. These T cells also recruit antitumor macrophages to the tumor microenvironment via CCL5/4-CCR5 and CCL3/CCL3L3-CCR1 signaling interactions. Furthermore, *IFNG*+ T cells activate cytotoxic T cells through CLEC2B/C/D-KLRB1 signaling, enhancing their tumor-killing capacity. Conversely, *CD163+* macrophages promote an immunosuppressive environment (Fig. S6d). They drive Treg differentiation via IL18-IL18R1 signaling, leveraging Tregs’ ability to suppress antitumor immunity. Additionally, *CD163+* macrophages inhibit Tph cell activation, a key player in antitumor immunity, through CD274-PDCD1 signaling and suppress cytotoxic *CD8*+ T cells via CD86-CTLA4 signaling. Collectively, these findings elucidate the opposing roles of these two key immune cell subpopulations.

### IFN-γ from T cell subpopulations induces differentiation in seminoma

While we have identified tumor-intrinsic genes associated with stemness in seminoma, it remains unclear whether external factors within the tumor microenvironment influence seminoma differentiation. To explore this possibility, we performed NicheNet analysis on the single-cell dataset, comparing differential gene expression between metastatic and non-metastatic seminomas. This analysis revealed that IFN-γ, secreted by T cells, exerted the strongest influence on these differentially expressed genes (Fig. 6a). Among the IFN-γ-responsive genes, *IFI6*, *IFI35*, *IFIT1*, *IFIT3*, *IRF1*, *ISG15* and *STAT1* were identified. Expression analysis confirmed that these genes were predominantly expressed in the non-metastatic seminoma subpopulations (Fig. 6b–h). Furthermore, these genes exhibited a significant positive correlation with pseudotime scores, indicating their association with tumor cell differentiation (Fig. 6i).

To validate the role of IFN-γ in promoting seminoma differentiation, we cultured the Tcam-2 seminoma cell line with IFN-γ. Upon IFN-γ treatment, stemness-related markers were significantly downregulated, while genes associated with IFN-γ stimulation were upregulated (Fig. 6j, k).

To further investigate whether the previously identified stemness-related genes (*DPPA4* and *PSMA7*) could sustain seminoma cell stemness and counteract IFN-γ-induced differentiation, we overexpressed *DPPA4* and *PSMA7* individually in Tcam-2 cells and compared their response to IFN-γ treatment with that of control cells (Fig. 6l, m). While IFN-γ treatment induced a dramatic reduction of stemness markers in control groups, the response was significantly blunted in the overexpression group compared to the NC group. These results suggest that *DPPA4* and *PSMA7* may confer resistance to external differentiation cues, protecting seminoma cells from the effects of IFN-γ-induced differentiation (Fig. 6n).

Collectively, these findings highlight distinct tumor and microenvironment components associated with tumor stemness and metastasis. A molecular panel was generated using genes associated with these components (Table S1). The panel successfully identified seminoma patients with a higher risk of disease progression, achieving an AUC of 0.737 for progression-free interval (PFI) and 0.706 for disease-free interval (DFI) (Fig. S7a–d). The implement of this molecular signature would facilitate improved patient stratification and ultimately advanced precision medicine.

## Discussion

In our study, we identified key genes associated with tumor stemness and metastasis. *DPPA4* and *PSMA7* were found to maintain stemness and promote metastasis, while overexpression of *PAGE5* and *SAT1* induced differentiation and inhibited metastasis. Within seminoma microenvironment, we also identified two key immune subpopulations which are associated with disease progression. Collectively, we identified a molecular panel based on these analyses, offering important insights to the risk stratification and contribute to the precision medicine.

Our findings suggest that the seminoma subgroup associated with metastasis exhibits molecular features more similar to those of embryonal carcinomas or atypical seminomas, indicating potential biological heterogeneity within seminomas [34]. Consistently, *MAGEA4*, a classical seminoma marker, was significantly downregulated in metastatic seminomas compared to non-metastatic cases, with even lower expression observed in embryonal carcinomas (Fig. S8) [35]. This reduction suggests that metastatic seminomas may deviate from the classical seminoma phenotype. In contrast, *PODXL*, a marker associated with atypical seminomas, showed markedly higher expression in both metastatic seminomas and embryonal carcinomas (Fig. S8) [36, 37].

Testis is a well-known immune-privileged organ. Consistently, our analysis revealed that immunosuppressive *CD163*+ macrophage was the only myeloid cell type in normal testis (Fig. S9a–c). Moreover, we observed that the expression of M2-related genes and signatures was significantly elevated in metastatic seminomas compared to their non-metastatic counterparts, approaching levels observed in the normal testis (Fig. 5p, q and S9d–g). These findings suggest that metastatic seminomas may exploit immunosuppressive cell populations to promote tumor progression and facilitate metastasis.

Interestingly, although the normal testis microenvironment lacks T cells, we identified diverse T cell subpopulations in the seminoma immune microenvironment. Notably, previously well-known tumor-associated immune cells, such as cytotoxic *CD8*+ T cells and Tregs, showed no correlation with metastasis in our study. Instead, seminoma malignancy was specifically associated with *IFNG*+ T cells. Furthermore, IFN-γ secreted by *IFNG*+ T cells in the non-metastatic seminoma microenvironment effectively counteracts this immunosuppressive state, restoring the functional capabilities of immune cells that attack tumor cells. Importantly, *IFNG*+ T cells were absent in more aggressive NSGCTs, further supporting their negative association with malignancy across TGCT subtypes (Fig. S4b). These findings provide a rationale for exploring IFN-γ-based therapies in clinical settings.

Seminoma originates from gonocytes, which have the potential to further differentiate into spermatogonial stem cells while retaining a degree of self-renewal capacity and stemness. Identifying genes that sustain stemness in seminoma may deepen our understanding of the physiological mechanisms governing gonocyte stemness and differentiation. Through single-cell RNA-seq analyses, we discovered key regulators of stemness in seminoma, including *DPPA4* and *PSMA7*. *DPPA4* is a nuclear factor known to play a critical role in maintaining pluripotency by modifying chromatin structure and regulating the transcription of pluripotency-associated genes [38, 39]. It is widely implicated in sustaining the undifferentiated state of embryonic and germline stem cells [40]. On the other hand, *PSMA7*, an essential component of the ubiquitin-proteasome system, has been implicated in stemness in few literatures [41]. However, its specific role in maintaining stemness in seminoma remains to be thoroughly investigated.

However, several limitations should be acknowledged in our study. First, the comparisons were made between tumors that had already progressed to metastatic disease and those that had not, making it unclear whether the observed molecular differences were present at diagnosis or emerged during disease progression. Prospective studies that follow patients from initial diagnosis are needed to determine whether these molecular and immune signatures can reliably predict aggressive behavior or metastatic potential. In addition, while our single-cell RNA sequencing revealed important transcriptional states, it did not address the underlying clonal structure of seminomas. Without lineage tracing or mutation-based clonal analysis, it remains unclear whether the aggressive, stem-like tumor cells identified represent fixed clonal lineages or reflect transient phenotypic states influenced by the microenvironment. Future studies incorporating clonal dynamics and prospective designs will be critical to fully elucidate the mechanisms driving seminoma metastasis and improve the clinical translation of our findings.

Building upon prior single-cell studies, our analysis includes multiple patient samples rather than being limited to individual cases or small cohorts [28, 42]. This broader dataset enables a more comprehensive and representative view of inter-patient heterogeneity and the molecular characteristics that differentiate metastatic from non-metastatic seminomas, including enhanced stemness and increased migratory and proliferative capacities. We identified molecular markers that facilitate risk stratification based on metastatic potential, which may support more individualized management strategies for seminoma patients.

## Methods and materials

### Patient sample collection process

Human primary seminoma samples were collected from the Fudan University Shanghai Cancer Center and Ninth People’s Hospital, Shanghai Jiao Tong University School of Medicine. All cases were pathologically confirmed by the Department of Pathology. This study was approved by the Ethics Committee of Shanghai Ninth People’s Hospital, affiliated with Shanghai Jiao Tong University School of Medicine, and the Research Ethics Committee of Shanghai Cancer Center, Fudan University. Written informed consent was obtained from all participants.

### Clinical follow-up of cohort patients

All ten patients included in our study underwent regular clinical monitoring for at least 28 months following orchiectomy, with a median follow-up of 31 months (range: 28–37 months). Clinical evaluations consisted of contrast-enhanced CT scans of the chest, abdomen, and pelvis, accompanied by serum tumor-marker assessments (β-hCG, AFP, LDH). By the end of the follow-up period, all seven patients in the non-metastatic cohort remained free of radiological and biochemical evidence of recurrence or metastasis. Therefore, the patients selected for our single-cell RNA sequencing analyses reliably represent seminoma cases with or without metastases, allowing us to investigate potential molecular markers differentiating tumors with distinct metastatic potentials.

### Preparation of single-cell suspensions from tissue samples

For our single-cell RNA sequencing experiments, we carefully selected non-necrotic tissue samples from both the tumor periphery (invasive front) and the central regions of each primary seminoma specimen [43]. These tissues were combined, processed simultaneously, and used to generate mixed single-cell suspensions. Tissue samples were preserved on ice in the sCelLive^TM^ Tissue Preservation Solution (Singleron Bio Com, Nanjing, China) within 30 mins of resection. The specimens were washed with Hanks Balanced Salt Solution (HBSS) and then digested with 2 ml sCelLive^TM^ Tissue Dissociation Solution (Singleron) in the Singleron PythoN™ Automated Tissue Dissociation System (Singleron) at 37 °C for 15 mins. The resulting cell suspension was treated with GEXSCOPE^®^ red blood cell lysis buffer (Singleron) at 25 °C for 10 mins. The cells were then centrifuged at 500 × *g* for 5 min and resuspended in PBS. Cellular viability was assessed with trypan blue (Sigma, United States).

### Preparation of single-cell libraries

The single-cell suspension was adjusted to a concentration of 1 × 10^5^ cells/ml and loaded onto microfluidic devices using the Singleron Matrix^®^ Single Cell Processing System (Singleron). ScRNA-seq libraries were constructed using the GEXSCOPE^®^ Single Cell RNA Library Kits (Singleron) following the manufacturer’s protocol. The resulting libraries were diluted to 4 nM, pooled, and sequenced on an Illumina Novaseq 6000 instrument with 150 paired end reads.

### Processing, quality control and analysis of raw data

The CeleScope (v1.1.7) pipeline was used to process raw FASTQ files and generate a filtered gene-cell matrix. Briefly, low-quality reads were removed, poly-A tails and adapter sequences were trimmed using Cutadapt (v1.17), and reads were aligned to the GRCh38 reference genome with STAR (v2.6.1a). The software featureCounts (v2.0.1) was then used to generate the expression matrix. The gene expression matrix was imported into R using Seurat (v4.0.1). Genes expressed in fewer than three cells were excluded, and cell quality metrics, including gene counts, UMI counts, and mitochondrial transcript percentages, were calculated. Low-quality cells with more than 20% mitochondrial genes, fewer than 200 genes, or more than 4000 genes were removed. Gene expression data were normalized and scaled using the *NormalizeData* and *ScaleData* functions, respectively. Highly variable genes (top 2000) were identified using the *FindVariableFeatures* function, and batch effects were corrected with the *RunFastMNN* function from SeuratWrappers (v0.3.0). Cells were clustered based on the top 30 principal components using the *FindClusters* function in Seurat, and UMAP dimensional reduction was performed with the *RunUMAP* function.

### Public scRNA-seq datasets

Publicly available scRNA-seq datasets utilized in this study were obtained from the GEO database with the following accession numbers: GSE197778, GSE112013, GSE143356, GSE124263, GSE134144, and GSE120508.

### Differentiation-associated analysis

Monocle 2 (v2.18.0) was employed to construct the pseudotime trajectory. The top 2000 variable genes, identified using the *FindVariableFeatures* function from the Seurat package, were selected for cell ordering with the *setOrderingFilter* function. Dimensional reduction was performed using the *reduceDimension* function using the DDRTree method. CytoTRACE (v0.3.3) was used to infer the differentiation trajectory in tumor cells.

### Pathway enrichment analysis

Enrichment analysis was conducted on the Metascape platform using default settings (https://metascape.org/gp/index.html#/main/step1). Gene set enrichment analysis (GSEA) was performed with the R package clusterProfiler (v3.18.1), utilizing gene sets from the Molecular Signature Database (MSigDB).

### TCGA and survival analysis

Expression and clinical data from the TCGA TGCT datasets were retrieved using the R package TCGAbiolinks. Tumor samples were scored with the singscore (v1.10.0) package using specific gene sets. Patients were stratified into high-risk and low-risk groups based on scores, determined with the *surv_cutpoint* function from the survminer package (v0.4.9). Survival curves were generated using the Kaplan-Meier method with the *survfit* function from the survival package (v3.2-7) and visualized using the *ggsurvplot* function. Receiver operating characteristic (ROC) curve was created using survivalROC (v1.0.3).

### Analysis of intercellular interactions

CellChat (v1.1.3) was employed with default parameters to analyze interactions between cell clusters. NicheNet (v1.1.1) was utilized to identify potential ligands influencing seminoma metastasis and phenotypes.

### Tumor microenvironment (TME) subtype classification

To assess the TME subtypes in our seminoma samples and relate them to previously established TME classifications, we adopted the analytical framework described by previous publications [32, 33]. Briefly, single-cell RNA sequencing data from each tumor sample were aggregated into pseudobulk profiles. For each pseudobulk sample, we calculated single-sample gene set enrichment analysis (ssGSEA) scores for 29 TME-related pathways, as defined in the published literature [32].

By referencing TCGA samples with annotated TME subtypes, the TME subtype of each sample was identified based on its ssGSEA profile, allowing classification into four major TME subtypes: Immune-Enriched (IE), Fibrotic (F), Desert (D), and Immune-Enriched/Fibrotic (IE/F, mixed type).

### Real-time PCR

Total RNA was extracted from cell lines using RNAiso Plus (TaKaRa, Japan), and cDNA was synthesized using the PrimeScript^®^ RT Reagent Kit with gDNA Eraser (TaKaRa, Japan). Target sequences were amplified via real-time PCR using TB Green Premix Ex Taq II on the Roche LightCycler 480 Real-Time PCR system. Primers used for gene expression quantification are listed in Table S2. Relative mRNA levels were calculated using the comparative threshold cycle (2^−ΔΔCT^) method, with *RPS29* serving as the reference gene.

### Immunohistochemistry

Formalin-fixed paraffin-embedded (FFPE) sections were collected. IHC staining was used to evaluated the protein expression levels. Primary antibodies used in this study are listed in Table S3. H-score was calculated by multiplying the percentage of stained cells and staining intensity according to previous publication [44]. The intensity scores included 0 (no evidence of staining), 1 (weak staining), 2 (moderate staining), 3 (strong staining).

### Cell culture

The human embryonal carcinoma cell line NCCIT (ATCC, CRL-2073) and the seminoma cell line Tcam-2 (Otwo, Wuhan, China) were used in this study. NCCIT cells were maintained in RPMI-1640 medium supplemented with 10% fetal bovine serum (FBS), while Tcam-2 cells were cultured in high-glucose DMEM supplemented with 10% FBS. All cells were cultured in an incubator at 37 °C in a humidified atmosphere containing 5% CO_2_.

### Generation of stable cell lines

ShRNAs targeting human *DPPA4* and *PSMA7* were designed using the WI siRNA selection program (http://sirna.wi.mit.edu), with sequences provided in Table S2. The shRNA sequences were cloned into the pGreenPuro-Dual vector. Human *SAT1* and *PAGE5* were also cloned into the pGreenPuro-Dual vector. Viral supernatants were collected and used to transduce target cells. Stable cell lines were selected using 3 µg/mL puromycin.

### Cell growth analysis

Cell growth was monitored using the Incucyte Live-Cell Analysis System (Sartorius, USA) following the manufacturer’s protocol. Briefly, NCCIT and Tcam-2 cells were seeded at a density of 3000 cells per well in 96-well plates and observed for 120 h. Growth curves were constructed from data collected at 4-h intervals during imaging.

### Incucyte 96-well scratch wound migration assay

Wounds were introduced into confluent NCCIT and Tcam-2 cell monolayers using the Incucyte 96-well WoundMaker tool. The plates were then placed in the Incucyte Live-Cell Analysis system, where live-cell images were captured every 4 h over a 48 h period. The confluence rate was quantified using the integrated analysis software of the Incucyte Live-Cell Analysis system.

### Sphere formation assay

A total of 50,000 cells were plated in 6-well ultra-low attachment plates (Corning, USA) containing DMEM/F12 medium supplemented with 20 ng/mL EGF and 10 ng/mL bFGF. Images were captured using a microscope (Olympus, Japan) and analyzed with ImageJ software (v1.53).

### Statistical analysis

Statistical analyses were conducted using R (v4.0.1) and GraphPad Prism (v9.5.0). Data normality was assessed with the Shapiro-Wilk test. Welch’s t-test was used for normally distributed data, while the Wilcoxon rank-sum test was applied to non-normally distributed data. Results are presented as mean ± SD for normally distributed data and as median with interquartile range (IQR) for non-normally distributed data. All experiments were repeated at least three times. Prior experiments with comparable methodologies were used to determine sample sizes. A P value of <0.05 was considered statistically significant (*P < 0.05, **P < 0.01, ***P < 0.001, ****P < 0.0001).

## Supplementary information

Figure S1

Figure S2

Figure S3

Figure S4

Figure S5

Figure S6

Figure S7

Figure S8

Figure S9

Table S1

Table S2

Table S3

Supplementary legends

## Data Availability

The raw sequence data of scRNA-seq reported in this paper have been deposited in the Genome Sequence Archive in National Genomics Data Center, China National Center for Bioinformation/Beijing Institute of Genomics, Chinese Academy of Sciences (GSA-Human: HRA009794/HRA005800) that are publicly accessible at https://ngdc.cncb.ac.cn/gsa-human.
