# Supplementary figures and images for: Single-cell analysis unravels divergent gene signatures shaping seminoma stemness and metastasis
Source: Cell Death Discov. 2025 Nov 7;11:514. doi: 10.1038/s41420-025-02802-4 (PMC12594921; doi:10.1038/s41420-025-02802-4)

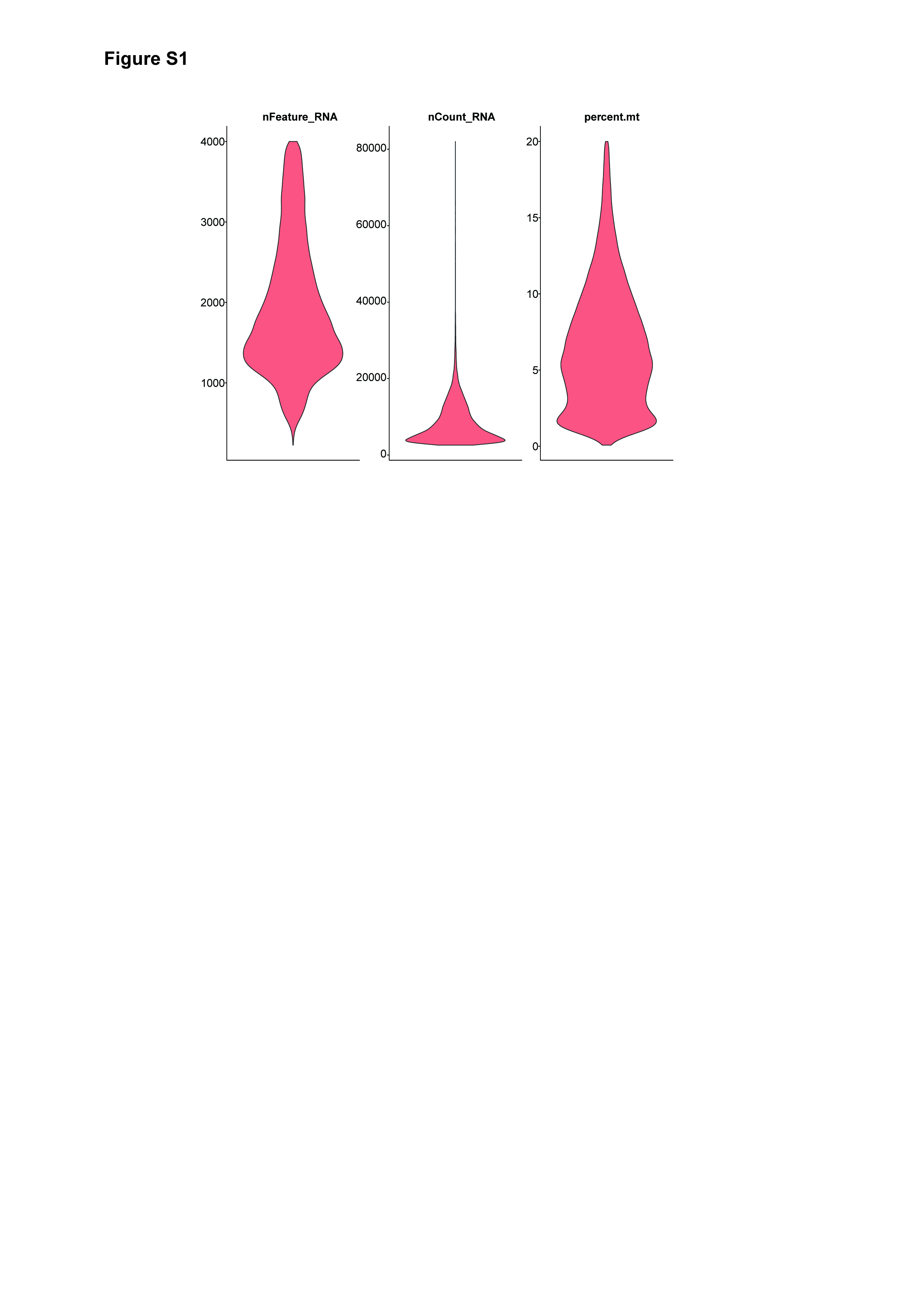

Supplement: Supplementary file 1 — Figure S1 [file 41420_2025_2802_MOESM1_ESM.tif]

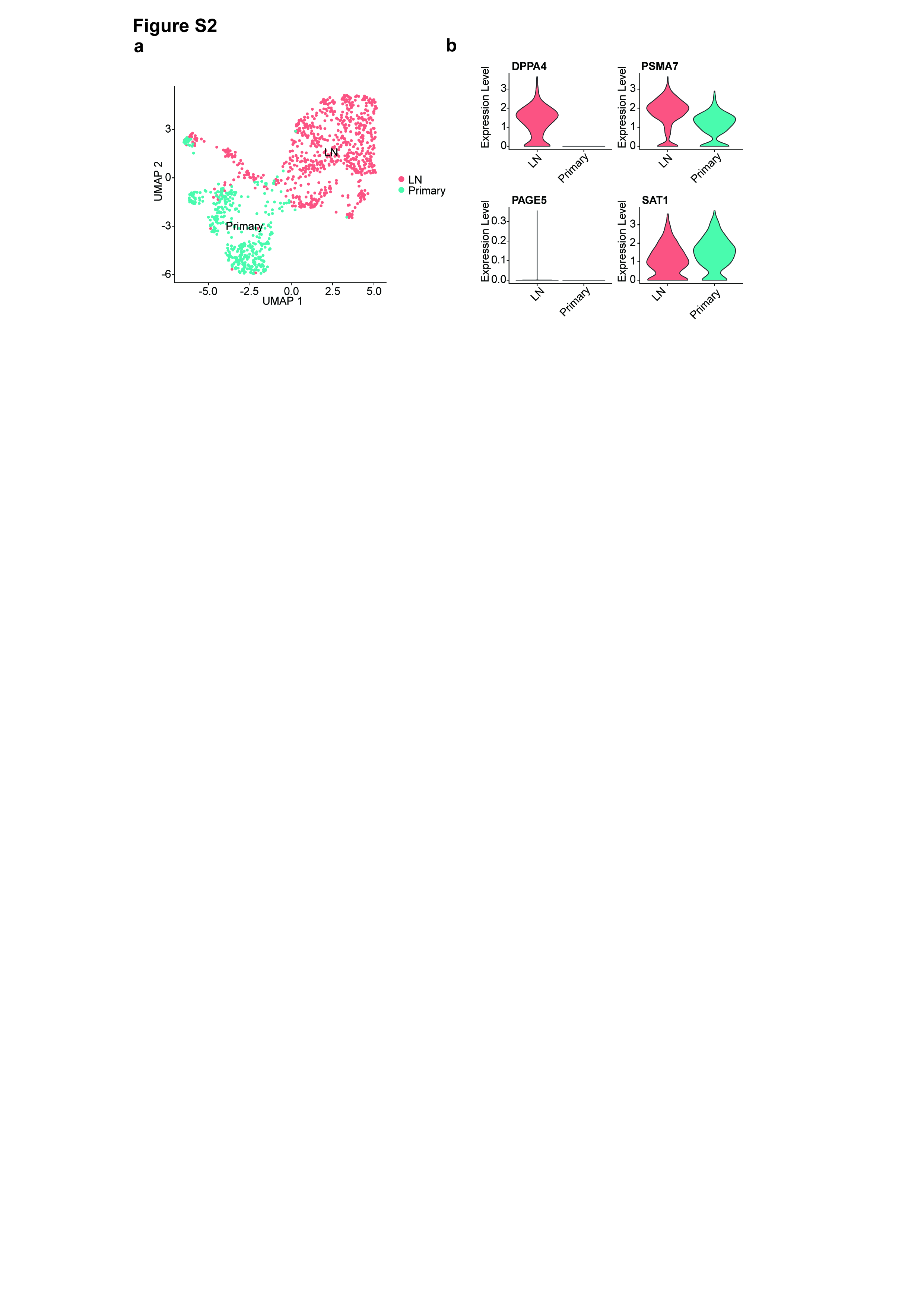

Supplement: Supplementary file 2 — Figure S2 [file 41420_2025_2802_MOESM2_ESM.tif]

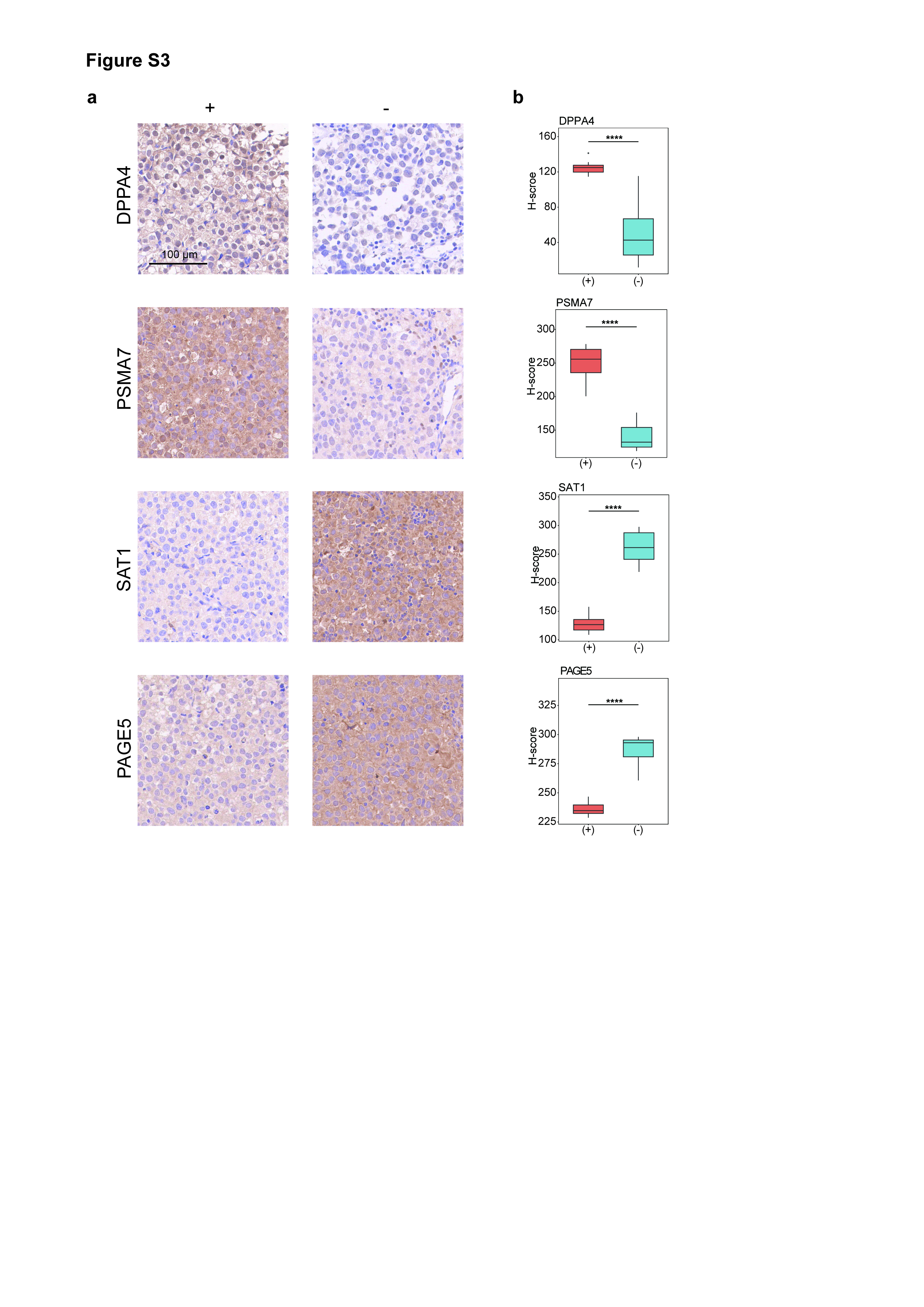

Supplement: Supplementary file 3 — Figure S3 [file 41420_2025_2802_MOESM3_ESM.tif]

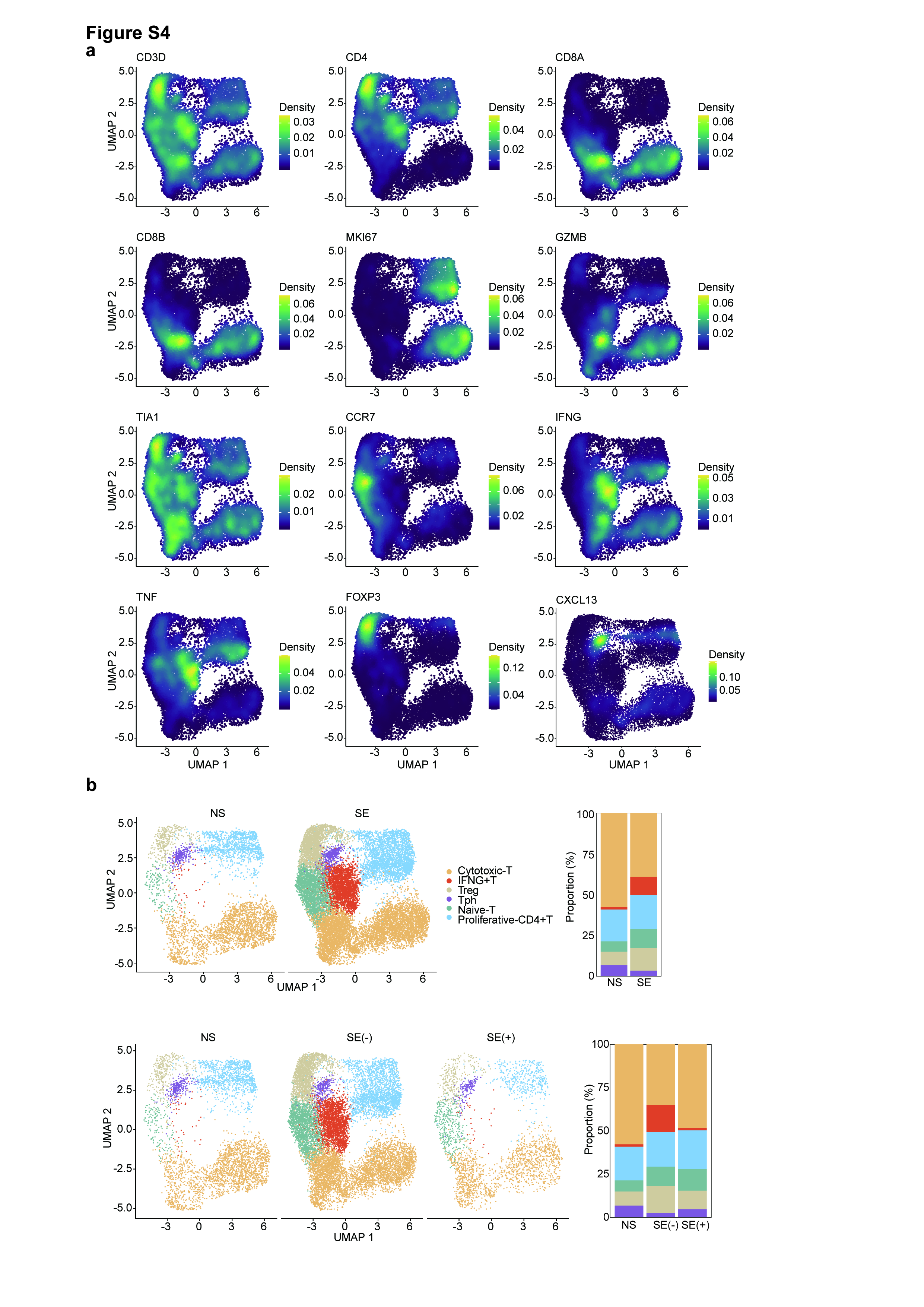

Supplement: Supplementary file 4 — Figure S4 [file 41420_2025_2802_MOESM4_ESM.tif]

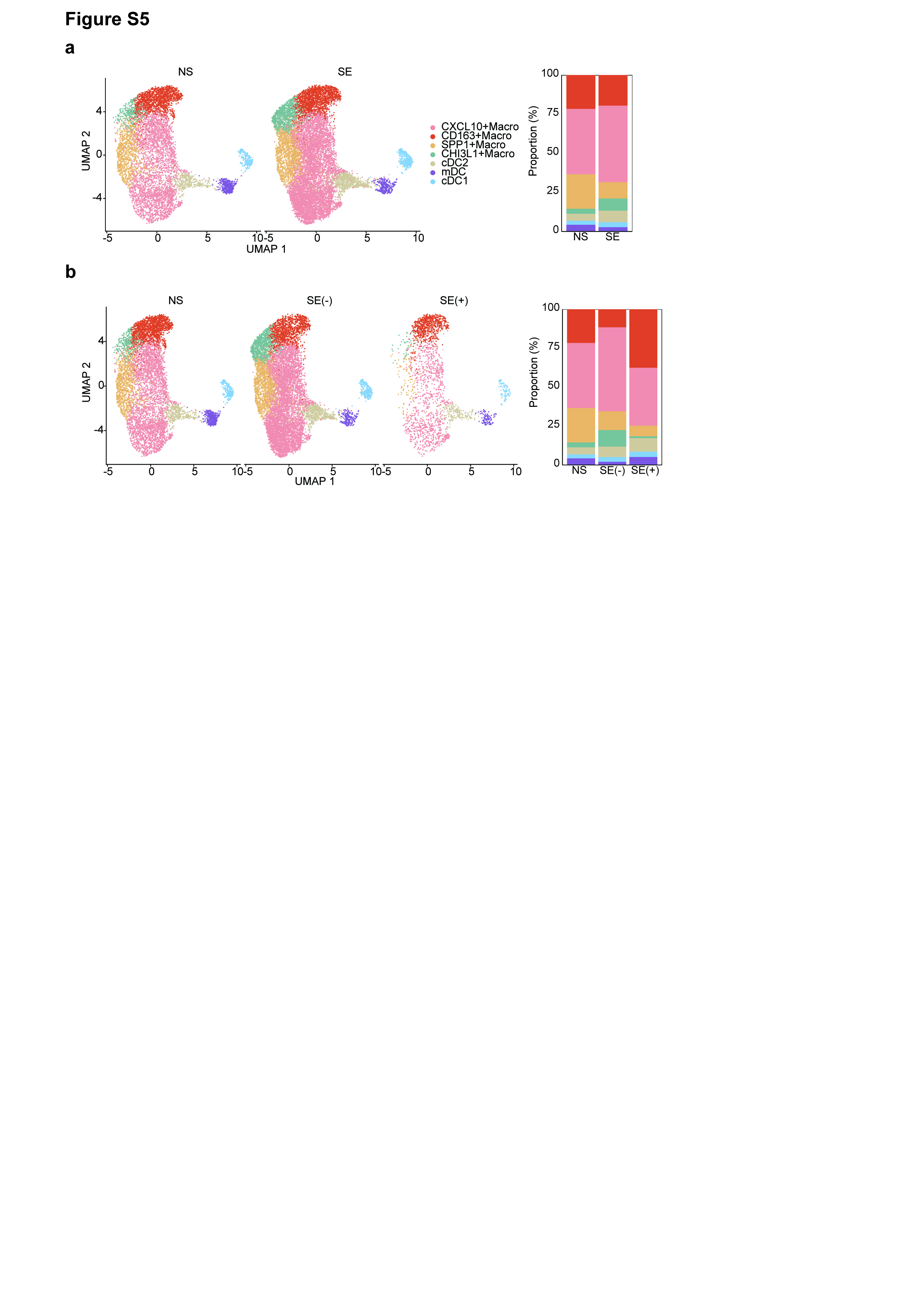

Supplement: Supplementary file 5 — Figure S5 [file 41420_2025_2802_MOESM5_ESM.tif]

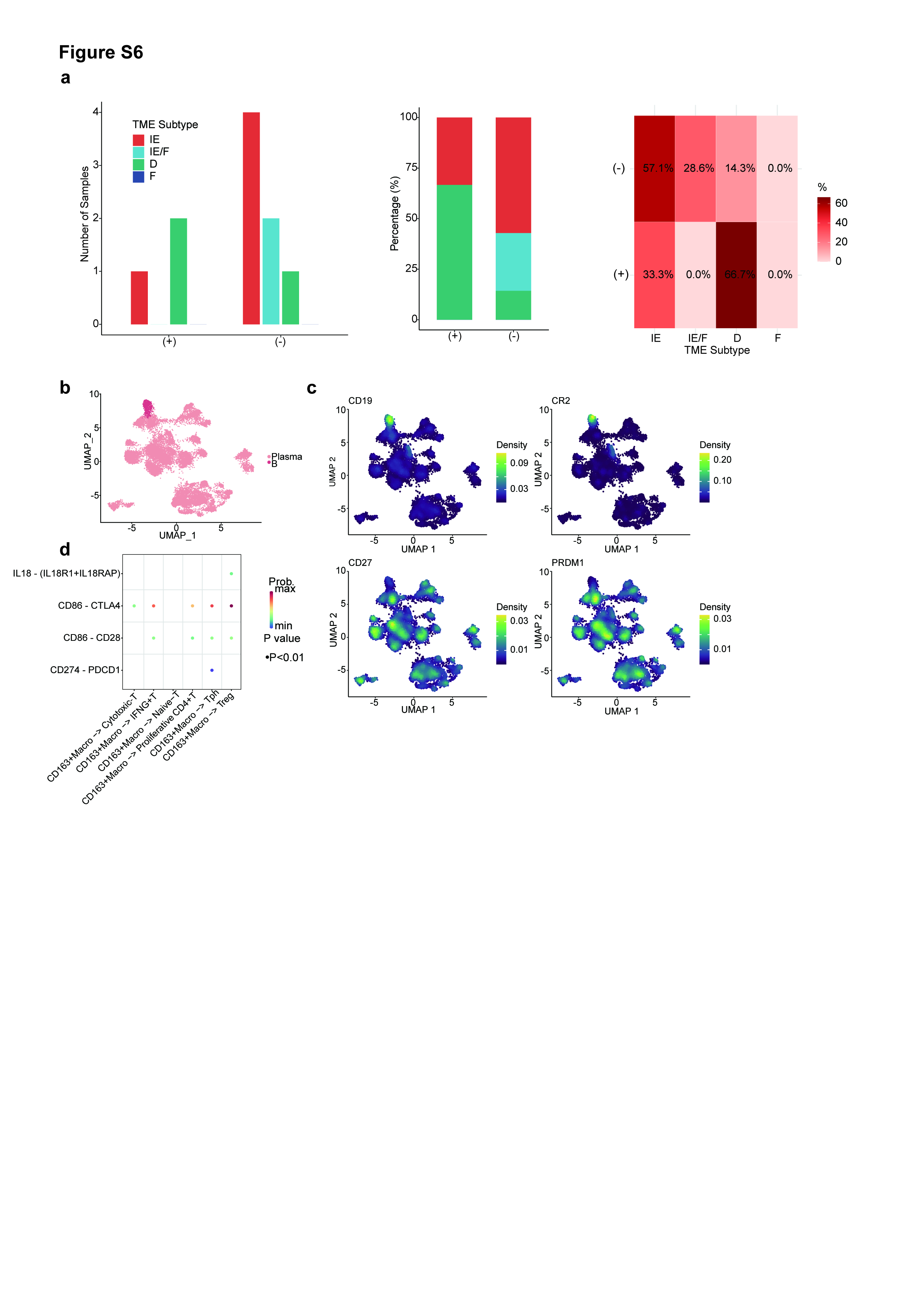

Supplement: Supplementary file 6 — Figure S6 [file 41420_2025_2802_MOESM6_ESM.tif]

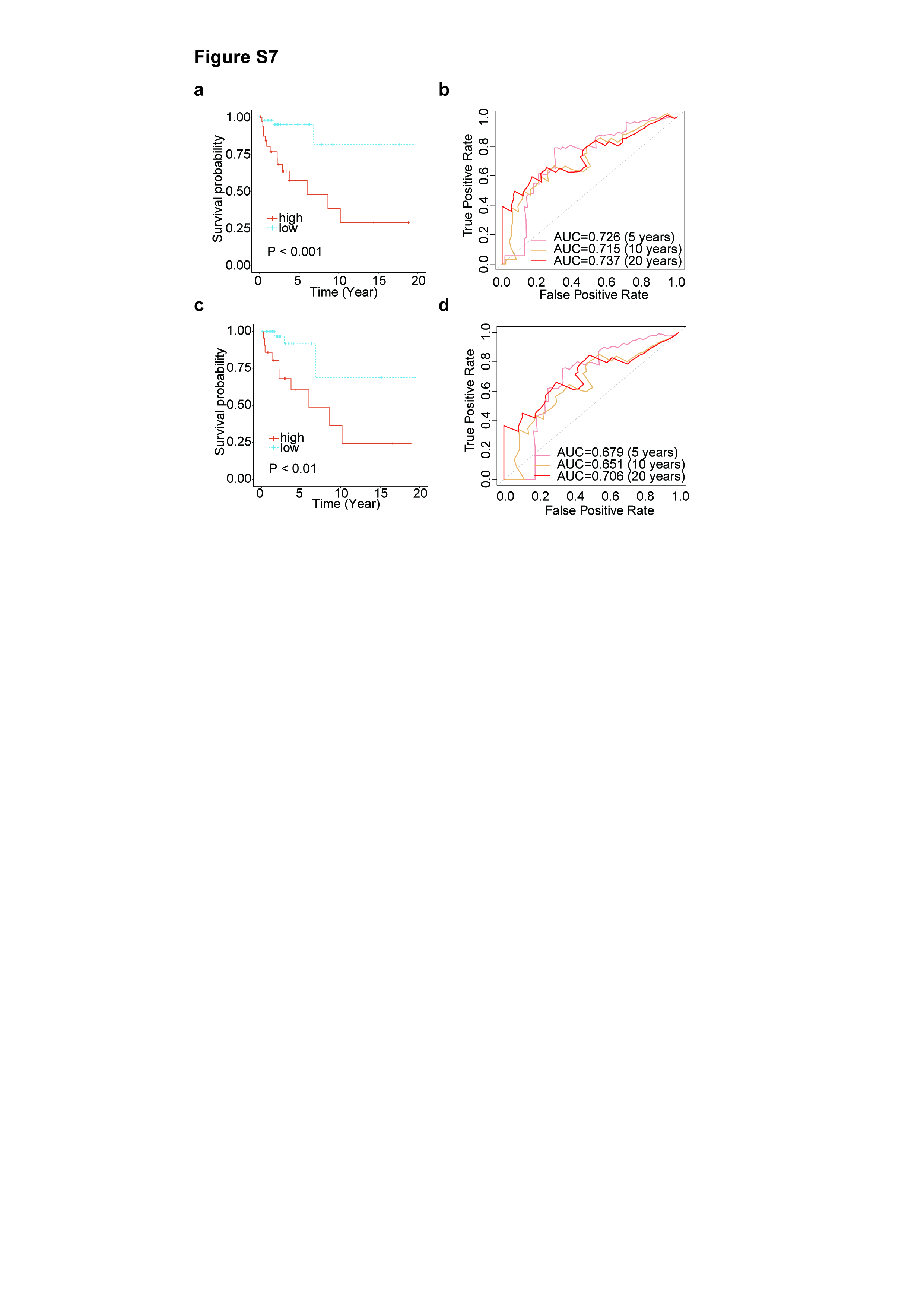

Supplement: Supplementary file 7 — Figure S7 [file 41420_2025_2802_MOESM7_ESM.tif]

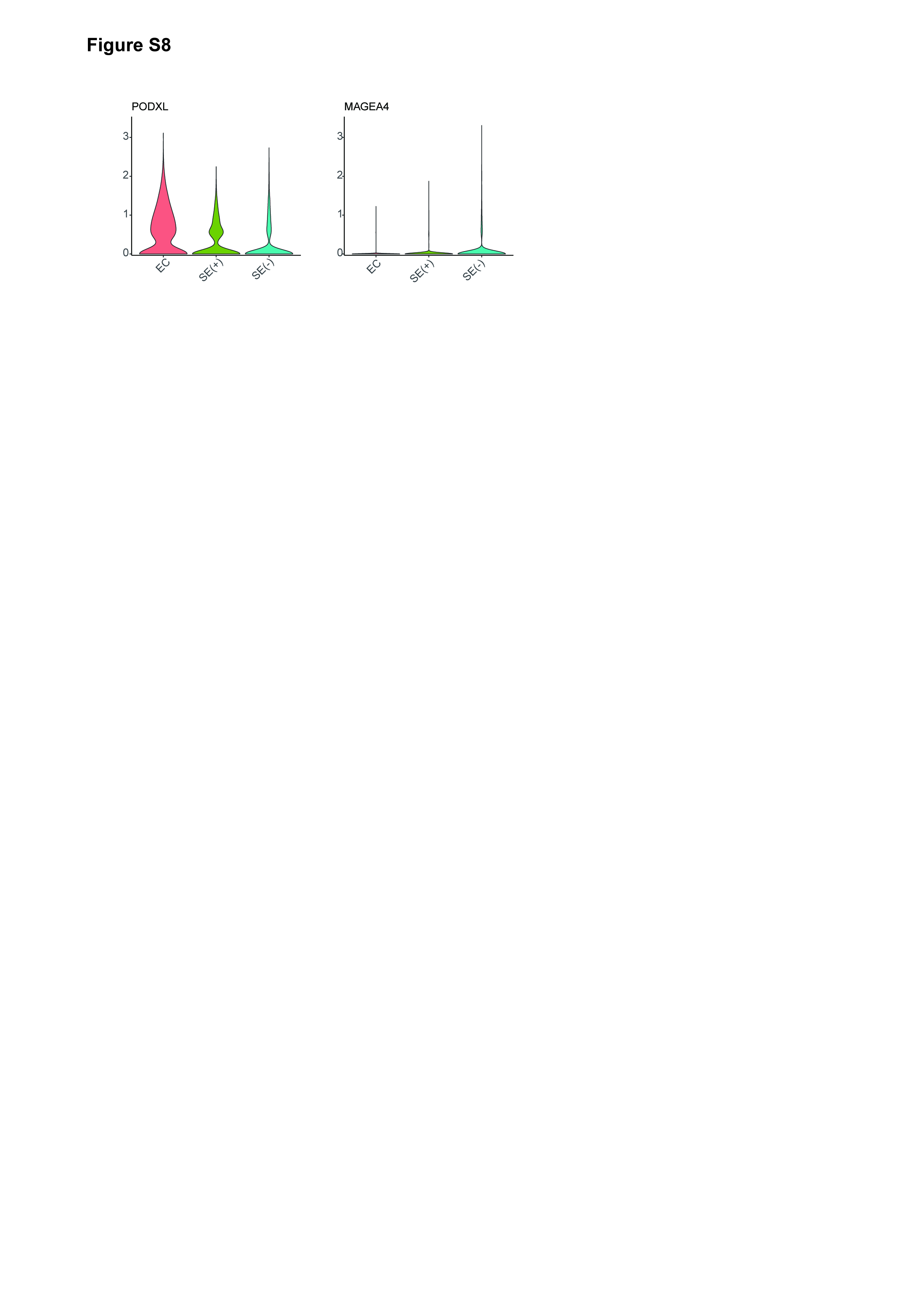

Supplement: Supplementary file 8 — Figure S8 [file 41420_2025_2802_MOESM8_ESM.tif]

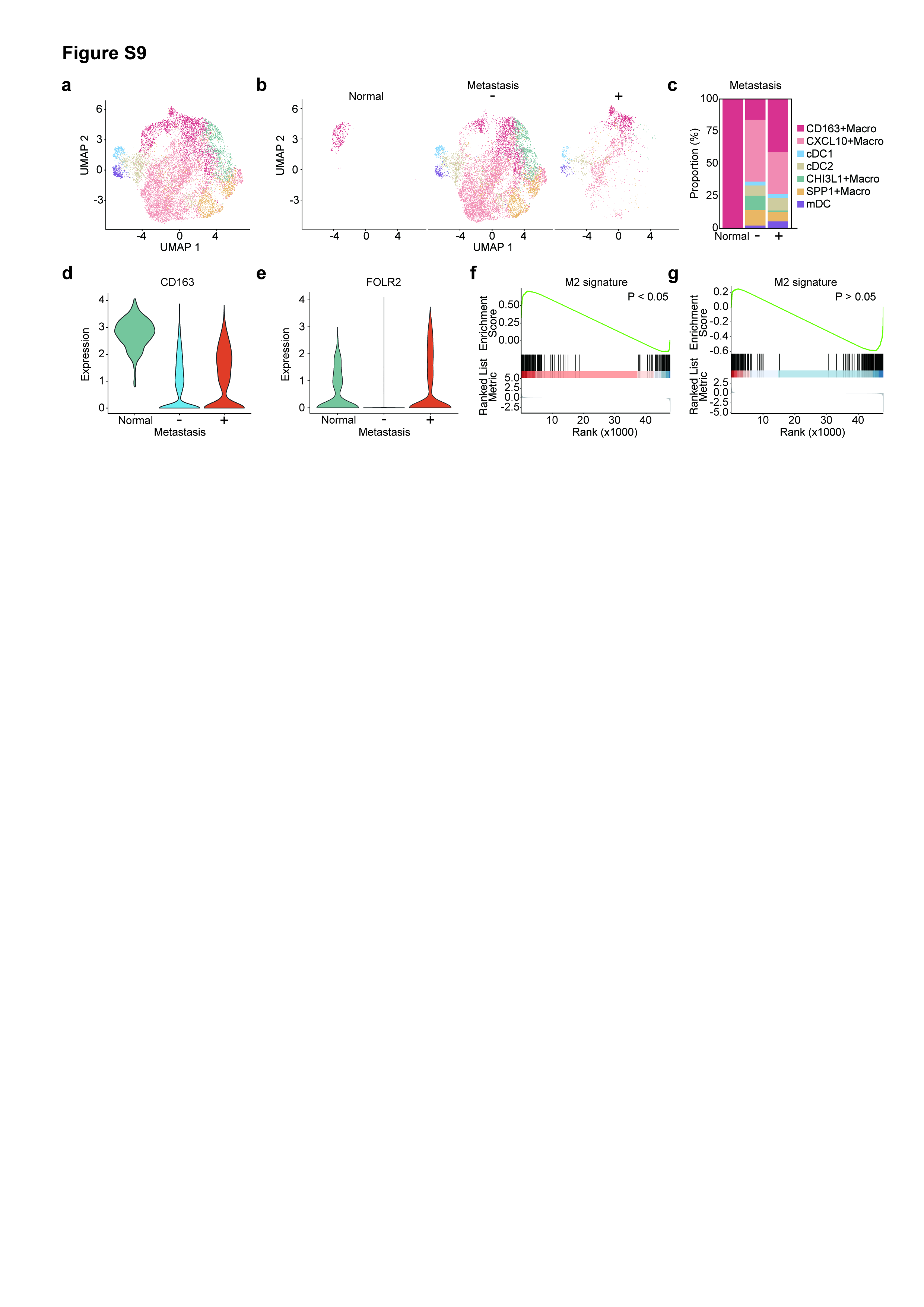

Supplement: Supplementary file 9 — Figure S9 [file 41420_2025_2802_MOESM9_ESM.tif]
