# Supplementary material for: Single-cell analysis unravels divergent gene signatures shaping seminoma stemness and metastasis
Source: Cell Death Discov. 2025 Nov 7;11:514. doi: 10.1038/s41420-025-02802-4 (PMC12594921; doi:10.1038/s41420-025-02802-4)
Supplement: Supplementary file 13 — Supplementary legends [file 41420_2025_2802_MOESM13_ESM.docx]

**Supplementary legends**

**Figure S1. ScRNA-seq quality control.**

**Figure S2. Analysis of public scRNA-seq dataset**

**(a)** UMAP plot showing paired primary and matched lymph-node metastasis tumor cells. **(b)** Violin plots comparing the expression levels of DPPA4, PSMA7, SAT1, and PAGE5 in tumor cells from the two sites.

**Figure S3. IHC analysis of DPPA4, PSMA7, SAT1, and PAGE5 expression in seminoma tissue samples from our cohort**

**(a)** Representative IHC staining images showing protein expression of DPPA4, PSMA7, SAT1, and PAGE5 in metastatic and non-metastatic seminoma tissue samples (scale bar: 100 µm). **(b)** Box plots comparing H-scores of each marker between metastatic and non-metastatic seminoma tissues.

**Figure S4. Identification of T cell subsets**

**(a)** UMAP plots showing the canonical markers of T cell subsets. **(b)** UMAP plots and stacked bar plot showing different cell subpopulations among different tumor subtypes (SE: seminoma; NS: NSGCT).

**Figure S5. Comparison of myeloid cell subsets**

**(a)** UMAP plots and stacked bar plot showing the distribution of myeloid cell subsets in seminomas and NSGCTs (SE: seminoma; NS: NSGCT). **(b)** UMAP plots and stacked bar plot comparing myeloid cell subsets in metastatic and non-metastatic seminomas and NSGCTs.

**Figure S6. TME classification and cell-cell communication analysis**

**(a)** Distribution of TME subtypes in metastatic and non-metastatic seminoma (IE: Immune-Enriched, F: Fibrotic, D: Desert and IE/F: mixed). **(b)** UMAP plot highlighting B and plasma cells. **(c)** UMAP plots showing the canonical markers of B/Plasma cell subsets. **(d)** Cell-cell interactions in metastatic seminomas.

**Figure S7. Molecular panel identification**

**(a)** Kaplan-Meier survival plot showing that the molecular panel correlates with worse PFI. **(b)** The ROC analysis for PFI. **(c)** Kaplan-Meier survival plot showing that the molecular panel correlates with worse DFI. **(d)** The ROC analysis for DFI.

**Figure S8. Expression analysis using markers of atypical seminoma**

**Figure S9. Comparison with normal testis microenvironment**

**(a)** UMAP plot of myeloid cells from normal testis and seminoma datasets. **(b)** UMAP plot showing different myeloid cell composition in normal testis, non-metastatic and metastatic seminoma datasets. **(c)** Bar plot summarizing the proportions of myeloid cell populations. **(d)** Violin plot comparing *CD163* expression in myeloid cells from normal testis, non-metastatic and metastatic seminomas. **(e)** Violin plot comparing *FOLR2* expression in myeloid cells from normal testis, non-metastatic and metastatic seminomas. **(f)** GSEA plot showing the enrichment of an M2-related gene signature in myeloid cells from normal testis compared to myeloid cells from non-metastatic seminoma. **(g)** GSEA plot showing no enrichment of an M2-related gene signature in myeloid cells from normal testis compared to myeloid cells from metastatic seminoma.

**Table S1. Molecular panel generated in this study**

**Table S2. Primers used in this study**

**Table S3. Antibodies used in this study**
